# Nickel affects xylem Sap RNase a and converts RNase A to a urease

**DOI:** 10.1186/1471-2229-13-207

**Published:** 2013-12-09

**Authors:** Cheng Bai, Liping Liu, Bruce W Wood

**Affiliations:** 1 Laboratory of Pests Comprehensive Governance for Tropical Crops, of Ministry of Agriculture, Hainan Laboratory for Monitoring and Control of Tropical Agricultural Pests, Hainan Engineering Research Center for Biological Control of Tropical Crops Diseases and Insect Pests, Environment and Plant Protection Institute, Chinese Academy of Tropical Agricultural Sciences, Haikou, Hainan 571101, China; 2 United States Department of Agriculture, Agricultural Research Service, Southeast Fruit and Tree Nut Research Laboratory, Byron, GA 31008, USA

**Keywords:** Urea, Pecan, Xylem sap, Ribonuclease, Nickel, Nitrogen cycling

## Abstract

**Background:**

Nickel (Ni) is an essential micronutrient; however, its metabolic or physiological functions in plants and animals are largely uncharacterized. The ribonucleases (RNase, e.g., RNase A) are a large family of hydrolases found in one form or many forms facilitating nitrogen (N) cycling. It is currently unknown how either a deficiency or excess of Ni influences the functionality of ribonucleases, like RNase A. This is especially true for perennial crops possessing relatively high Ni requirements.

**Results:**

We report that the 'rising’ xylem sap of pecan [*Carya illinoinensis* (Wangenh.) K. Koch, a long-lived tree] at bud break contains a 14 kDa RNase A (aka, RNase 1), which amount has a 33% greater in Ni-deficient as in Ni-sufficient trees when exposed to Ni ions exhibits ureolytic activity. The homologous 13.4 kDa bovine pancreatic RNase A likewise exhibits ureolytic activity upon exposure to Ni ions. Ni therefore affects enzymatic function of a typically non-metalloenzyme, such as it transforms to an enzyme capable of hydrolyzing a linear amide; thus, converting an endonuclease esterase into a urease.

**Conclusions:**

We conclude that Ni potentially affects the level and activity of RNase A present in the spring xylem sap of pecan trees, and probably in other crops, it has the same influence. The catalytic property of RNase A appears to shift from a nuclease to a urease relying on Ni exposure. This is suggestive that RNase A might possess novel metabolic functionality regarding N-metabolism in perennial plants. The ability of Ni to convert the activity of plant and animal RNase A from that of a ribonuclease to a urease indicates a possible unrecognized beneficial metabolic function of Ni in organisms, while also identifying a potential detrimental effect of excessive Ni on N related metabolic activity if there is sufficient disruption of Ni homeostasis.

## Background

Nickel appears to be essential for life. Lower plants require Ni as an essential co-factor for many metalloenzymes [1], and higher plants require Ni as a co-factor for urease (EC 3.5.1.5, urea amidohydrolase) [2-4]. The roles of Ni in animal metabolism and physiology are largely uncharacterized; although, it appears to be essential, yet specific essential roles continue to evade elucidation [5]. The relatively broad adverse physiological impact of severe Ni deficiency in both plants and animals hints of multiple essential roles.

Urease is one of the few metalloenzymes known to require Ni for activity. The essential role for Ni in plants is partially based on the discovery that highly functional urease typically requires Ni ions. Urease is ubiquitous in higher plants because of its unique role in N metabolism for hydrolyzing urea, a linear amide, to NH_4_^+^ and CO_2_. Ureases vary in molecular size and number of subunits, depending on biological source. Most forms are relatively large proteins possessing small subunits [e.g., jack bean urease (a 580 kDa hexamer with identical subunits of 90,970 Da, and containing two Ni ions per subunit) [6] and urease from *Klebsiella aerogenes* (a Ni-containing multicomponent urease, 224 kDa; subunits of 72, 11 and 9 kDa; about two Ni ions per 72 kDa subunit)] [7]. Nickel is therefore important in plant metabolic processes, especially when rapid growth requires relatively large amounts of chemically reduced-N as NH_4_^+^.

The ability of organisms to metabolize and recycle N is requisite for life. Cycling involves many enzymes, with ribonucleic acid depolymerases (ribonucleases, or RNases), and their homo- and orthologues, playing a major role in breakdown and recycling of N-containing ribonucleic acids (RNAs). Prominent among the RNase forms is RNase A (RNase, EC 3.1.27.5), a highly durable protein ubiquitous in plants and animals [8,9]. RNase A is a non-metalloenzyme that cleaves single-stranded RNA at the 3′-end of pyrimidine residues and degrades RNA into 3′-phosphorylated mono- and oligo-nucleotides. This relatively small enzyme (13,473 Da for bovine pancreatic RNase A) is a monomer that forms a dimer (comprises of both a relatively large and a smaller component) upon concentration in mild acid. RNase is a highly stable enzyme, maintaining enzymatic integrity as an endonuclease esterase under conditions that cause most enzymes to lose functionality. The ribonuclease superfamily consists of many forms, homologues and orthologues, possessing novel biological activity based on ribonuclease homology [10,11]. The RNase A molecule can bind Ni ions [12,13], but it is unknown whether this affects hydrolase activity and catalyzing function.

Many enzymes exhibit limited dual catalytic activity, catalyzing more than one reaction type. Structural similarities between RNase A and urease molecules indicate that RNase A might be capable of hydrolyzing linear amides, such as urea, if Ni ions modify its molecular structure. If the Ni complex exhibits ureolytic activity then it might serve as an alternative means for ensuring timely N-metabolism in organisms [14]. An example might be the mobilization and conversion of organic-N within xylem sap of perennial plants as they transition from dormancy to active growth when Ni is in xylem sap.

Xylem sap of perennial plants can contain ribonucleases [15], but it is unknown whether they are present in long-lived perennials, such as pecan [*Carya illinoinensis* (Wangenh.) K. Koch]. Species possessing relatively high Ni requirements might benefit from Ni-associated ureolytic activity when reduced-N is, or is about to be, in high demand due to rapid growth of canopy organs during early spring [16]. It is presently unknown whether RNase A occurs in pecan xylem sap, and if so, whether it possesses ureolytic activity. It is also unknown whether RNase A from animals also exhibits ureolytic activity in presence of Ni. We therefore investigated these possibilities and found that xylem sap of pecan trees at time of spring bud break does indeed contain RNase A, and that exposed to Ni ions potentially converts pecan RNase A and bovine pancreatic RNase A to a urease.

## Results

**Figure 1:**
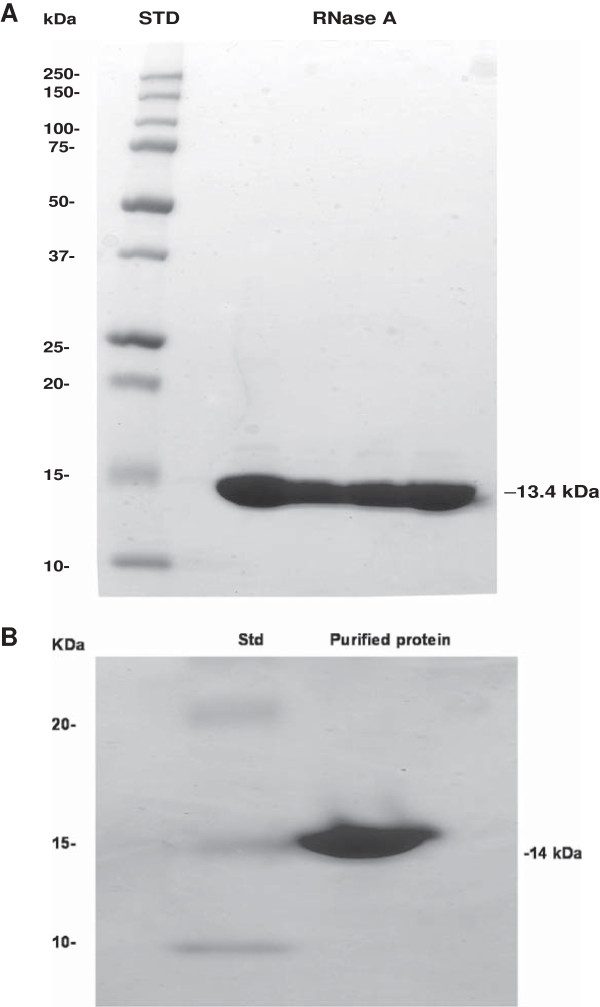
**Sodium dodecyl sulfate-polyacrylamide gel electrophoresis of bovine pancreatic and pecan xylem RNase A. (A)** Bovine pancreatic RNase A. Approximately 200 μg of protein was loaded. STD: Standard (loaded: 12 μl). Numbers in the left and right columns of the figure indicate molecular mass. **(B)** Purified pecan urease. The 14-kDa enzyme has been purified to homogeneity. Numbers in the left column and right side of the figure indicate molecular mass in kDa. The migration distance on the SDS-PAGE gel indicates the size of protein. Std = Protein Standards.

Pecan xylem sap at time of bud break contained a 14 kDa protein (Figure 1A). Sodium dodecyl sulfate-polyacrylamide gel electrophoresis (SDS-PAGE) indicated that this protein possesses a molecular mass (M_r_) similar to purified 13.4 kDa bovine pancreatic RNase A (Figure 1B; from USIB Corporation, Cleveland, OH, USA). The primary structure of pancreatic RNase A from different sources is known [17]. Purified xylem sap protein exhibits a single protein band of 14 kDa (Figure 1B) and was only about 33% as abundant in the xylem sap of 'Ni-deficient’ as in 'Ni-sufficient’ trees (Table 1). Similarly, xylem sap Ni was 48.4 μg/L in 'Ni-sufficient’ trees and 12.8 μg/L in 'Ni-deficient’ trees (Table 1).

**Table 1:** Concentration RNase A in pecan xylem sap as a function of Ni nutritional status

**Tree Ni nutritional status**	**Xylem sap Ni (μg/L)**	**RNase A (mg/L)**
Ni-sufficient	48.4a^*z*^	8.4a
Ni-deficient	12.8b	2.9b

^*z*^Means are statistically different by ANOVA at P < 0.0004 when followed by different letters.

The ureolytic activity of freshly purified 14 kDa xylem sap protein exhibits 371 units/mg protein (Table 2) compared to 25 units/mg for the crude xylem protein. The specific activity of the purified 14 kDa protein was 223 units/mg, which compares to 589 units/mg of purified jack bean urease (590 kDa) and purified bovine pancreatic RNase A (13.4 kDa) (Table 2). The 14 kDa xylem sap protein therefore exhibits roughly the same specific activity, as does bovine pancreatic RNase A, and about 40% that of jack bean ureases (Table 2).

**Table 2:** Ureolytic activity of purified pecan xylem sap RNase A compared to that of jack bean urease and bovine pancreatic RNase A

**Sample**	**Activity (Unit/ml)**^***a***^	**Specific activity (Unit/mg protein)**^***a***^
Purified bovine pancreatic RNase A (13.4 kDa)	179.9 ± 15.7	179.9 ± 15.7
Purified jack bean urease (590 kDa)^b^	589.0 ± 64.1	589.0 ± 64.1
Crude xylem sap proteins	24.93 ± 2.04	80.41 ± 6.56
Purified xylem sap 14 kDa protein	371.25 ± 17.33	222.75 ± 10.44
Buffer control	0	0

^a^Activity and Specific Activity is mean values (± SD) of three replicate assays; urease catalyzes the hydrolysis of urea to ammonia and carbon dioxide.

^b^The molecular mass for native jack bean urease is from 480 kDa to 590 kDa^13^ and calculated size is about 540 kDa for the hexamer.

**Figure 2:**
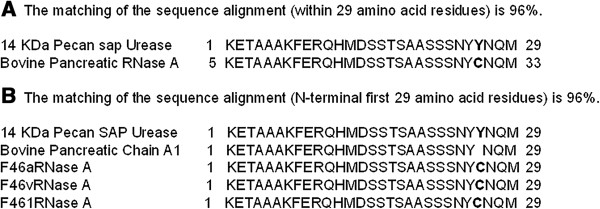
**The N-terminal sequence and alignment of 14-kDa pecan sap RNase A with reported proteins. (A)** 14-kDa pecan sap RNase A. The matching of the sequence alignment (within 29 amino acid residues) is 96%. **(B)** Bovine pancreatic RNase A. The matching of the sequence alignment (N-terminal first 29 amino acid residues) is 96%.

**Figure 3:**
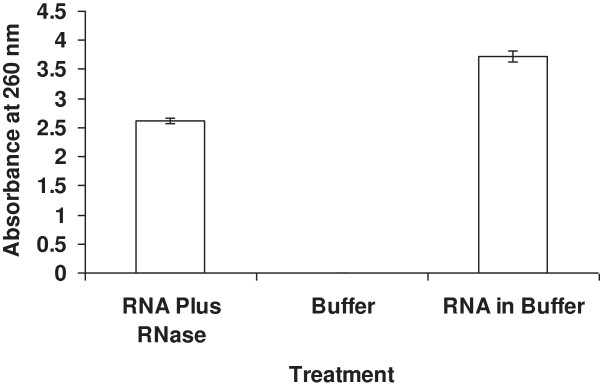
**Cleavage of RNA with bovine pancreatic 13.4 kDa protein.** Cleaving RNA with the pecan sap 14 kDa RNase A protein produces a similar graph.

**Figure 4:**
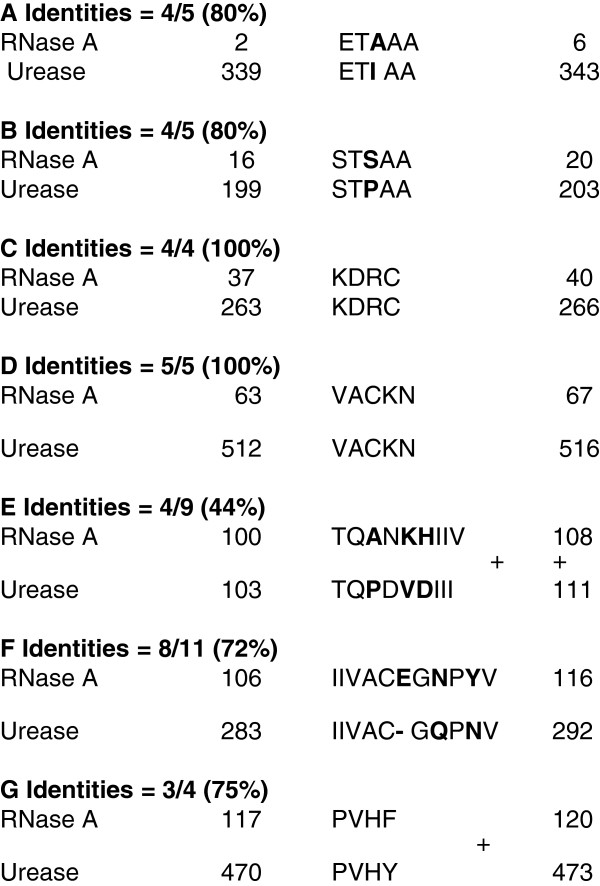
**Alignment BLAST/NCBI: Search results for short, nearly exact matches of bovine pancreatic RNase A (13,473 Da; total 124 amino acid residues) and urease (total 567 amino acid residues) from *Psychrobacter cryohalolentis *K5. A**. Identities = 4/5 (80%); **B**. Identities = 4/5 (80%); **C**. Identities = 4/4 (100%); **D**. Identities = 5/5 (100%); **E**. Identities = 4/9 (44%); **F**. Identities = 8/11 (72%); **G**. Identities = 3/4 (75%).

**Figure 5:**
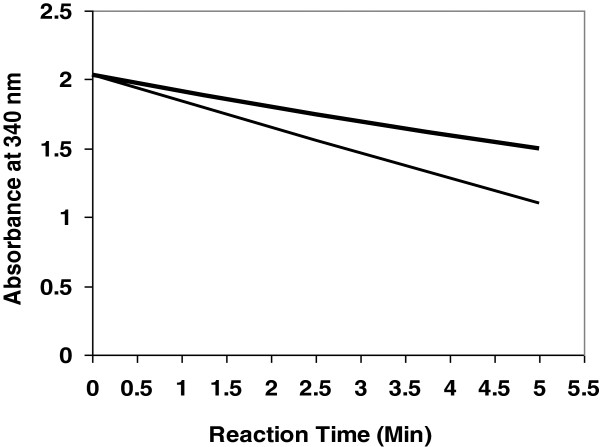
**Bovine pancreatic RNase A catalyzing the hydrolysis of urea.** Net absorbance at 340 nm was scanned after adding bovine pancreatic RNase A [6.7 μg protein (upper line) and 20 μg protein (lower line)] into the reaction mixture for urease activity assay. Data indicates a positive correlation between the amounts of RNase A added to the reaction mixture and the breakdown of urea. A very similar graph was obtained with pecan sap RNase A catalyzing the hydrolysis of urea.

Sequence determination (BLAST Search, NCBI) confirms that the N-terminal amino acid sequence of the 14 kDa pecan xylem sap ureolytic protein (Figure 2) is 96% identical to that of bovine pancreatic RNase A (RNase 1) and bovine pancreatic chain A 1 (or F46aRNase A, F46vRNase A, and F46lRNase A) [18]. This slight M_r_ difference between these two RNases might be due to a transacted form of certain amino acid residues in the xylem sap protein, or a few amino acid residues longer for the bovine RNase A. This indicates that the 14 kDa ureolytic protein from pecan xylem sap is likely RNase A, and this is further confirmed in that this xylem sap protein also exhibits RNase A activity (Figure 3). Similarly, the purified bovine 13.4 kDa RNase A and pecan RNase A did indeed possessed nuclease activity in that it cleaved RNA (Figure 3). The BLAST (NCBI) database [18] indicates that the amino acid sequence of bovine pancreatic RNase A (total 124 amino acid residues) is 44-100% matched in seven molecular regions with urease (total 567 amino acid residues; Figure 4) from *Psychrobacter cryohalolentis* K5 [8]; however, there is no apparent sequence matching with jack bean urease [18]. The Ni dose used was positively correlated with the ureolytic activity of RNase A from either bovine or pecan xylem sap, with both activated by relatively high concentration of Ni ions (Table 3). Bovine pancreatic RNase A catalyzes the hydrolysis of urea (Figure 5), with reaction time being a critical factor for hydrolysis—i.e., the longer of the reaction time, the greater the urea hydrolysis. Urea degrading activity increases as amount of RNase A increases in the reaction mixture (Figure 5).

**Table 3:** Effect of Ni ions on ureolytic activity of pecan xylem sap and bovine pancreatic RNase A

**Nickel (II) Nitrate concentration *In Prepared Solution (μM)***	***In reaction system (μM/ml)***	**Relative ureolytic activity of pecan Xylem Sap RNase A ** **(%)**	**Relative ureolytic activity of bovine Pancreatic RNase A ** **(%)**
0	0	100	100
333	0.003	150	143
1,000	0.010	188	174
3,333	0.033	251	238

## Discussion

### RNase A in pecan xylem sap

Ribonuclease activity, molecular mass and amino acid sequence analysis identified a 14 kDa RNase A present as a component of the late winter xylem sap of a long-lived perennial tree crop at a time when the pecan tree is transitioning from dormancy to initiation of early spring growth. This is a time when the tree requires access to considerable amounts of chemically reduced N for growth of new tissues and organs in preparation for the upcoming growing season. The Ni nutritional status influences the concentration of xylem sap RNase A. A biochemical consequence of Ni deficiency in plants is a potential reduction in RNase A within spring xylem sap. This deficiency potentially limits N cycling and metabolism at a critical time, in which perennial plants are preparing for rapid growth, by impairing RNA catabolism in apoplastic space.

### Activity of the pecan Ni-RNase A complex

It is of physiological significance that when pecan RNase A is exposed to Ni it exhibits ureolytic activity. The ureolytic activity of this xylem sap 14 kDa Ni-RNase A complex is similar to that of the bovine pancreatic Ni-RNase A complex, but only about 40% as great as that of jack bean urease. While there is a theoretically possibility that the purified xylem sap RNase A protein contained a urease contaminate, the rigor of the purification protocol is unlikely to have left such a contaminate. However, further study is required in order to definitively eliminate such a possibility. The results are suggestive that xylem sap RNase A plays a duel role in regards to early season N metabolism of perennial plants. In addition to a primary function of cleaving RNA, there is a putative secondary function of catabolizing urea in ascending xylem sap and associated apoplastic space. These activities are likely to vary as a function of sap Ni concentration. Whether or not this ureolytic activity actually occurs *in planta* remains to be determined. The presence of urea in the apoplast, from either urea uptake from soil by roots, or from catabolism of purine nucleotides, ureide pathway intermediates, or from arginine argues that small apoplastic proteins capable of converting urea to NH_4_ might prove metabolically important, especially during the time of transitioning from dormancy to active growth.

### The influence of Ni on RNase A

Our finding that the Ni-RNase A complex from sources as diverse as pecan and cattle exhibits substantial ureolytic activity naturally raises questions about its relevance to N metabolism naturally occurring in plants and animals. It appears that the nature of this ureolytic activity depends on cellular Ni status, which in turn should vary according to homeostatic processes and organismal exposure to Ni. Transition metal ions, such as Ni, elicit various enzymatic characteristics, including nucleophilic catalysis, electron transfer, and stabilization of protein structure. X-ray diffraction and three-dimensional structures of RNase A indicates the enzyme possess potential to bind Ni ions at His 105, where it acts as a nucleation site and causes a conformational change [19] capable of enabling significant ureolytic activity. Similarly, a plant urease from *Kiebsilla aerogenes* appears to possess a functional requirement for two Ni ions per active site [7], with Ni ions bound at Arg 70 and Gly 197 (amino acid residues) where Ni easily makes contact with urea substrate molecules [13].

Observations herein indicate that Ni binding to RNase A, normally a non-metalloenzyme, enables ureolytic activity; thus, the Ni-RNase A complex is functionally an alternative molecular form of urease, an essential Ni-metalloenzyme. This complex might therefore play a role in urea catabolism by perennial plant species, especially those like pecan that possess relatively high Ni requirements. A similar role might also exist in other plants and animals. While Ni possesses an essential metabolic role in plants and animals, that role is somewhat enigmatic. It now appears possible that in certain cases Ni could be influencing N cycling through its influence on RNase A. While many proteins can bind or contain Ni [20], this aspect of Ni nutritional physiology in has received little attention *in planta* or *in animal*.

Several enzyme systems in bacteria and lower plants require Ni. Examples include NiFe-hydrogenase, carbon monoxide dehydrogenase, acetyl-CoA decarbonylase synthase, methyl-coenzyme M reductase, superoxide dismutase, Ni-dependent glyoxylase, aci-reductone dioxygenase, and methyleneurease [1,21,22]. It is the activation of urease, to date, that is the most commonly recognized function of Ni in higher plants [22]. Ni can also replace Zn or Fe, and other transition metal ions, in certain other metalloenzymes of lower plants [1], and can probably do so to some degree in higher plants and animals. Circumstantial evidence indicates that ureide-transporting plant species, such as pecan, possess a higher Ni requirement than amide-transporting species [23]; thus, raising the possibility that ureide transporting species might possess enzymes, other than the standard ubiquitous urease, that require Ni for activation or for enhanced activity. Likely candidates are one or more enzymes affecting N metabolism; thus, raising the possibility, that RNase A possesses a dual role in cellular metabolism and that this role depends on endogenous bioavailability of Ni.

This potential ureolytic form of RNase A in plants and animals possibly affects N-metabolism via hydrolysis of either urea or RNA depending on Ni exposure. For example, in germinating rice seeds, Ni^2+^ exposure increases the levels of RNA by a decline in hydrolysis [24-26]; however, in eggplant ribonuclease activity is increased by Ni^2+^[26].

This is the first report of RNase A exhibiting urease activity; hence, we postulate that the reaction for urea hydrolysis in many organisms is as follows:

$${\left({\mathrm{NH}}_{2}\right)}_{2}\mathrm{CO}+H_{2}O\overset{\mathrm{Urease}\;\mathrm{or}\;\mathrm{RNase}\;A}{\underset{{\mathrm{Ni}}^{++}}{\to }}{\mathrm{CO}}_{2}+2{\mathrm{NH}}_{3}$$

It appears that not only does bovine pancreatic RNase A potentially possess duel functionality as urease when exposed to Ni ions, but that a pecan orthologue also functions as an urease within the early spring xylem sap of pecan when N reserves are being mobilize from storage tissues to emerging sinks. Urea is a catabolic product of ureide catabolism, e.g., allantoate and ureidoglycolate [27-29]; thus, the xylem sap, and affiliated apoplast, of many long-lived perennials may be a location contributing to the conversion of urea to NH_4_ in preparation for protein and nucleic acid anabolism because of imminent deployment of canopy organs. Both free and bound Ni readily transports in xylem vessels [30-32] and therefore potentially influences the nature of RNase A activity.

This discovery potentially broadens knowledge of possible secondary roles of RNase A in organisms in regards to N metabolism. Because several structural orthologues or paralogues of ribonucleases possess biological action [10,11], it is possible that Ni binding to these proteins also alters their activity; thus, highlighting new possibilities for metabolically important roles for Ni in higher organisms.

## Methods

### Plant materials

Three-year-old pecan seedlings, originating from seed of open pollinated 'Desirable’ cv. trees, were grown in pots within a greenhouse to produce two Ni nutritional classes of trees—'Ni sufficient’ (Ni^+^) vs. 'Ni deficient’ (Ni^-^), based on expression of morphological symptoms of Ni deficiency [11]. The Ni^-^ trees were produced from growing in a Tifton Loamy Sand soil. This soil often causes Ni deficiency symptoms in associated commercial pecan orchards. Ni^+^ trees from the same soil had prior season July leaf Ni concentrations of 1–4 μg^.^g^-1^ dry weight, which is considered to be within the 'sufficiency’ range for Ni in pecan. The Ni^-^ trees had Ni concentrations ≤ 0.8 μg^.^g^-1^ dry weight [33], which is low enough to trigger the appearance of easily visible morphology based symptoms.

Six specimens from each of the two 'Ni status classes’ were randomly chosen for study from a population of trees, based on expression of visual symptoms of Ni deficiency occurring at the time of spring bud break. Spring xylem sap was collected and analyzed from several trees exhibiting the two classes of Ni nutritional status. Collection was at bud break, with bud break defined as inner bud scale split of >50% of primary apical buds. Xylem sap was collected by vacuum extracted from stems severed at the root collar and again just below the apical tip; with the phloem and bark associated with the stem base peeled back to ensure that xylem sap was not contaminated with phloem sap. The base of severed stems was placed under vacuum and the exuding xylem sap dripped into 2 ml collection vials. Total Ni concentration of xylem sap of Ni^-^ and Ni^+^ trees was determined with xylem sap collected as described above, and quantified by ICP-MS [34].

### Extraction and purification of xylem sap urease

The xylem sap samples were diluted in Buffer E [25 mM 2-morpholineethanesulfonic acid (Mes), pH 6.2, 2.5 mM MnSO_4_, and 2.5 mM DTT] and centrifuged at 20,000 *g* for 1 h and 30,000 *g* for 1 h. This supernate was passed through a 0.22 μM membrane to remove impurities, and then twice extracted with chilled acetone (90%) to precipitate proteins. This precipitate was dissolved in Buffer E, with protein purification via ammonium sulfate fractionation, gel filtration chromatography and ion exchange chromatography according to previously reported methods [35]. Proteins were further purified by passing through ammonium sulfate fractionation (50%), filtering to exclude ≤ 10 kDa molecules (Amicon Centricon YM-10, Millipore Corporation, Bedford, Ma, USA), and finally via gel filtration (Superdex 200 10/300 GL column; controlled by ÄKTAbasic –Systems, Amersham Biosciences, Piscataway, NJ, USA) and ion exchange (Mono Q H/R 5/5 column; controlled by ÄKTAbasic –Systems) chromatography. Buffer E was used as solvents and NaCl was added into Buffer E for the salt gradient elution during purification [36]. Fractions with urease activity were pooled, desalted using a small P-10 column, and concentrated by centrifugation for 2 h at 5,000 *g* at 4°C with Amicon Centricon YM-10. The smaller molecules were removed because they contain phenolic substances that interfere with enzyme activity. Fresh purified enzyme was subsequently loaded on large (16 cm × 16 cm) gradient (8-16%) sodium dodecyl sulphate -polyacrylamide electrophoresis (SDS-PAGE) gels for purity analysis.

### RNase A source and activity

RNase A from bovine pancreas (90 units/mg protein) came from the USIB Corporation (Cleveland, OH, USA). Its purity was examined with SDS-PAGE (10-20% gradient gel, 16 cm × 16 cm in size) while controlling temperature (at 12–15°C) during electrophoresis. Purified protein was denatured in SDS-gel sample buffer and electrophoresed on a SDS-10 to 20% polyacrylamide gradient gel. The nuclease activity of RNase A from bovine pancreas was verified by suspending RNA (ribonucleic acid from baker’s yeast, *Saccharomyces cereviae;* Sigma, St. Louis, Mo, USA) in Buffer E [25 mM 2-morpholineethanesulfonic acid (Mes), pH 6.2, 2.5 mM MnSO_4_, and 2.5 mM DTT) [35]. RNase A (10 μL) was then added to the RNA suspension and the reaction mixture (2 mg/ml) incubated at 25°C for 1 h and then centrifuged (5,500_g_ at 4°C). The mixture was then filtered to exclude ≤ 50 kDa molecules (Amicon Centricon YM-50, Millipore Corporation, Bedford, Ma, USA). The fraction was rinsed with Buffer E, centrifuged (at 5,500_g_ at 4°C), and again filtered to exclude ≤ 50 kDa molecules. The > 50 kDa fractionation was suspended in Buffer E (2 ml) and the reaction monitored based on absorbance at 260 nm. Buffer E served as a blank and untreated RNA (2 ml) served as a positive control.

### Assay of urease activity in protein solutions with molecular mass ≥10 kD

Urease activity was described by Kaltwasser and Schlegel [36], but with slight modification [37]. The assay is completed as a coupled enzyme with Glu dehydrogenase. All chemical assay reagents were dissolved in 0.1 M potassium phosphate buffer (pH 7.6). The assay mix was 0.37 ml of 0.1 M potassium phosphate buffer (pH 7.6), 0.1 ml of 1.8 M urea, 0.1 ml of 0.025 M ADP, 0.2 ml of 0.008 M NADH, 0.1 ml of 0.025 M α-ketoglutarate before adding 0.1 ml of 50 units/ml Glu dehydrogenase and 5 μL of enzyme solution. The change in *A*_340_ at 25°C was recorded at 0.5, 1, 3, and 5 min. Urease of jack bean (*Canavalia ensiformis*; 29.5 units/mg; Sigma, St. Louis, Mo, USA) was used as a reference. A unit is defined as the amount of urease causing oxidation of 1 μM of NADH/min at 25°C, pH 7.6, in a coupled reaction using Glu dehydrogenase. Protein concentration was determined with the Bio-Rad protein assay with bovine serum albumin as standard.

### Effect of nickel ions on urease activity of RNase A

The purified RNase A solution (10 μl), either from bovine or pecan sap, was mixed with 10 μl of Ni-nitrate solution at different concentrations (0, 333, 1,000, and 3,300 μM, respectively) in a cuvette to give a final Ni concentration of 0, 0.0033, 0.0100, and 0.0333 μM/ml. After 2 min, 998 μl of chemical substrate solution was added and mixed, with measurement of urease activity determined as described above.

### Determination of the N-terminal amino acid sequence

Purified urease from pecan sap was denatured in SDS-gel sample buffer and electrophoresed on an SDS-10 to 20% polyacrylamide gradient gel and then the protein was transferred to a PVDF membrane. The amino acid sequence of N-terminals was determined by Edman degradation and performed by a Molecular Biology Resource Facility (University of Oklahoma Health Science Center, Oklahoma City, OK, USA).

## Abbreviations

RNase: Ribonuclease; N: Nitrogen; Ni: Nickel; SDS: Sodium dodecyl sulphate; PAGE: Polyacrylamide gel electrophoresis; PVDF: Polyvinylidene fluoride; MES: Morpholineethanesulfonic acid; DTT: Dithiothreitol; BLAST: The Basic Local Alignment Search; NCBI: The National Center for Biotechnology Information; STD: Standard; ADP: Adenine 5′-diphosphate; NADH: Nicotinamide adenine dinucleotide, reduced form; 2D gel: Two dimension gel.

## Competing interests

The authors declare that they have no competing interests.

## Authors’ contributions

CB collaborated in the designed the concept, conducted most experiments and contributed to the manuscript. LPL performed some experiments and participated in the interpretation of the results. BWW participated in the design of concept, Ni analysis, interpretation of results, and writing and editing the manuscript. All authors read and approved the final manuscript.
